# The mitochondrial genome of the land snail *Theba pisana* (Müller, 1774) (Stylommatophora: Helicidae): the first complete sequence in the genus *Theba*

**DOI:** 10.1080/23802359.2018.1491341

**Published:** 2018-07-27

**Authors:** Pei Wang, Shan-Ping Yang, Jun-Hong Lin, Ming-Zhe Zhang, Wei-Chuan Zhou

**Affiliations:** a Key Laboratory of Molluscan Quarantine and Identification of AQSIQ, Fujian Entry-Exit Inspection & Quarantine Bureau, Fuzhou, China;; b College of Plant Protection, Fujian Agriculture and Forestry University, Fuzhou, China;; c Zhejiang Academy of Science and Technology for Inspection and Quarantine, Hangzhou, China

**Keywords:** *Theba pisana* (Müller 1774), Helicidae, mitochondrial genome, phylogeny

## Abstract

The complete mitochondrial genome of the white garden snail *Theba pisana* (Müller, 1774) has been sequenced and annotated in this study. The entire circular genome is 14,795 bp in size and represents the first mitochondrial genome in the genus *Theba*, with two ribosomal RNA genes, 22 transfer RNA genes, 13 protein coding genes. All of genes are divided into two groups, including 24 genes on the majority coding strand (J strand) and others on the minority coding strand (N strand).The phylogeny supports the relationship of species in Helicidae.

A well-known agricultural pest *Theba pisana* (Müller, 1774) has become an invasive species in many countries worldwide (Odendaal et al. 2008). The snail not only seriously destroys legume crops, cereals, fruits, etc., but also spread schistosomiasis as an intermediate host for the terrestrial trematode parasite *Brachylaima cribbi* (Butcher and Grove 2005; Odendaal et al. 2008). Here, we sequenced the complete mitochondrial (mt) genome of this snail, which can offer more worthwhile information for phylogeny and be applied in molecular alignment and identification.

The complete mt genome was sequenced on the Illumina Hiseq 2500 platform at Berry Genomics, Beijing. Adult snail was collected from Ca’n Pastilla, Mallorca, Spain in 2013 (39°32′21″N, 2°42′59″E), and kept in 100% ethanol before being transferred to –20 °C for long-term preservation at the Herbarium of plant pests, Fujian Entry-Exit Inspection & Quarantine Bureau (FJCIQ), Fuzhou, Fujian, China. Total genomic DNA was extracted from the pedal muscle tissue of single individual using the DNeasy Blood and Tissue kit (Qiagen) according to the manufacturer’s instructions. The tRNA genes were identified with tRNAscan-SE Search Server v.1.21 (Lowe and Eddy 1997) and DOGMA (Wyman et al. 2004). The PCGs and rRNA genes were annotated by BLAST in Genbank with published available mitochondrial sequences of terrestrial snails (Deng et al. 2016; Wang et al. 2014; Yang et al. 2014). Phylogenetic analyses were performed using maximum likelihood (ML) method.

The entire circular genome was 14,795 bp in length, containing 13 protein coding genes, 22 transfer RNA genes, 2 ribosomal RNA genes (Genbank accession number MH362760). 24 genes were encoded on the majority coding strand (J strand) except other 13 genes (*tRNA^Gln^*, *tRNA^Leu(UUR)^*, *tRNA^Asn^*, *tRNA^Arg^*, *tRNA^Glu,^ tRNA^Met^, tRNA^Ser(UCN)^*, *tRNA^Thr^*, *ATP6*, *ATP8*, *ND3*, *COIII* and *SrRNA*) oriented on the minority coding strand (N strand). The nucleotide composition of the whole genome was biased towards adenine and thymine, accounting for 66.80%. All PCGs started strictly with ATN (one with ATC, two with ATG, two with ATA, and eight with ATT). Conventional stop codons TAA and TAG had been assigned to all of PCGs except *ND3* with a single T. The length of tRNA genes ranged from 49 to 68 bp. The length of *lrRNA* and *srRNA* were determined to be 979 and 698 bp, respectively.

The ML tree (Figure 1) presented 17 major clades containing the Bradybaenidae, Camaenidae, Hygromiidae, Helicidae, Polygyroidae, Urocoptoidae, Cerionidae, Succineidae, Orthalicidae, Clausiliidae, Achatinellidae, Pupillidae, Vertiginidae, Achatinidae, Agriolimacidae, Lymnaeidae and Aplysiidae. The five bradybaenid species and two camaenid species each formed a clade and the sister-group relationship between the two clades was also recovered. Four species in the Helicidae formed a monophyletic group. However, the phylogeny of Camaenidae, Helicidae and Bradybaenidae are complicated and have not been fully resolved; systematic and phylogenetic studies based on analyses of morphological and molecular markers have produced inconsistent results (Scott 1996; Wade et al. 2007; Hirano et al. 2014). More taxon sampling need to be prepared to assess the phylogenetic relationship of these three families.

**Figure 1.**
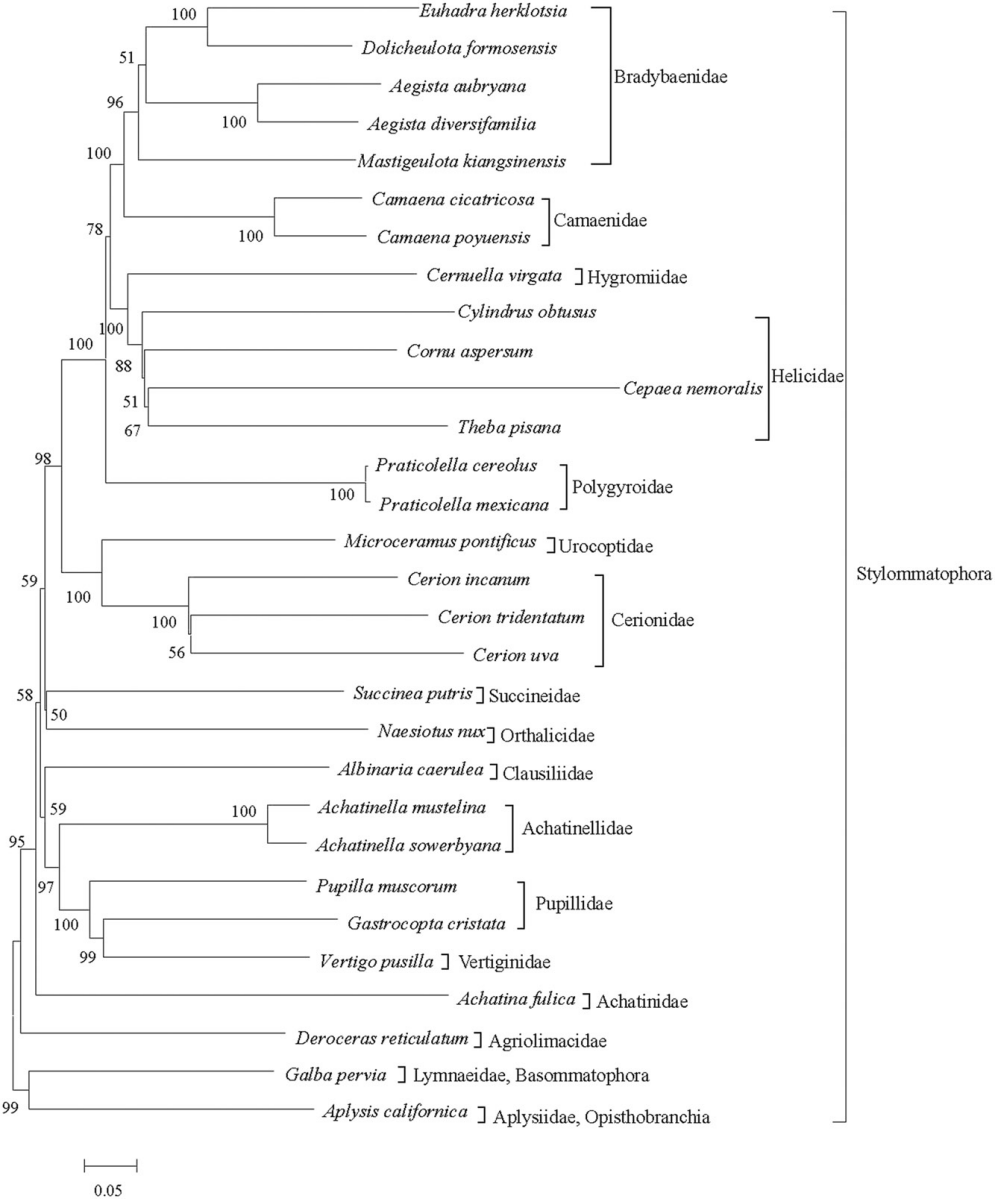
Phylogenetic tree inferred by ML method based on 13 protein genes. The tree is rooted with *Aplysis californica* and *Galba pervia*. Numbers on or under the nodes represent bootstrap values.
